# Negative Outcomes of Blepharoplasty and Thyroid Disorders: Is Compensation Always Due? A Case Report with a Literature Review

**DOI:** 10.3390/diseases12040075

**Published:** 2024-04-10

**Authors:** Beatrice Defraia, Martina Focardi, Simone Grassi, Giulia Chiavacci, Simone Faccioli, Gianmaria Federico Romano, Ilenia Bianchi, Vilma Pinchi, Alessandro Innocenti

**Affiliations:** 1 Forensic Medical Sciences, Department of Health Science, University of Florence, Largo Brambilla 3, 50134 Florence, Italy; beatricedefraia@gmail.com (B.D.); martinafocardi@gmail.com (M.F.); simone.grassi@unifi.it (S.G.); giuliachiavacci1@gmail.com (G.C.); simone.faccioli@unifi.it (S.F.); vilma.pinchi@unifi.it (V.P.); 2 Laboratory of Personal Identification and Forensic Morphology, Department of Health Sciences, University of Florence, Largo Brambilla 3, 50134 Florence, Italy; 3 Doctor in Medicine Specialized in Plastic Reconstruction and Aesthetic Surgery, Via Francesco Baracca, 1f, 50127 Firenze, Italy; gfredromano@hotmail.com; 4 Plastic Reconstructive Microsurgery, Careggi University Hospital, Largo Brambilla 3, 50134 Florence, Italy; a.innocenti@unifi.it

**Keywords:** blepharoplasty, thyroid diseases, plastic surgery, surgical malpractice, surgery complications

## Abstract

Background: Plastic surgery is one of the medical specialties with the highest risk of recurrent medical malpractice claims. The frequency of civil lawsuits represents an issue for the micro- and macro-economy of practitioners of these health treatments. This paper aims to discuss the medico-legal aspects and claim path in a case of a cosmetic blepharoplasty complicated by lagophthalmos wrongly related to the procedure but due to missed hyperthyroidism. Case Description and Literature Review: A 48-year-old woman who underwent cosmetic blepharoplasty with undiagnosed hyperthyroidism claimed that the lagophthalmos that occurred some months after the procedure was due to medical malpractice, due to an over-resection of the exuberant lower eyelid tissue. The review question was, “Are thyroid disfunctions usually considered contraindications to be communicated to patients who undergo blepharoplasty?”, and the databases MEDLINE via PubMed, Embase, Scopus, Ovid, ISI Web of Science, Cochrane, and Google Scholar were used. Results and Discussion: There were 21 eligible papers. The case highlights the importance and complexity of causal inference (such as unknown thyroid dysfunctions), related informed consent involving information on possible complications unrelated to malpractice, and guidelines recommending endocrinological consultation for cosmetic/functional blepharoplasty in patients at risk (e.g., female patients with a known history of thyroid disease).

## 1. Introduction

Blepharoplasty can be performed for cosmetic reasons or to amend functional impairments, like ptosis or herniated orbital fat [1,2,3,4]. It is a technically complex surgery since it can be sensitive to anatomical structures [5]. From a medico-legal point of view, plastic surgery is one of the medical specialties at the highest risk of recurrent medical malpractice claims [6]. In detail, among the procedures of plastic surgery, blepharoplasty and rhinoplasty are the most exposed to medical malpractice claims [7,8]. The frequency of medical malpractice claims is relevant because civil lawsuits are an issue not only from a micro- and macroeconomic point of view: Studdert et al. found that recurrent claims are associated with a higher risk of reducing the volume of treated cases and of ceasing practice [6]. When a medical malpractice claim is evaluated, finding and proving the cause of a negative outcome is the most sensible and complex step. In particular, in cases of negative outcomes of surgical procedures, compensation is generally due if the plaintiff creates a rebuttable presumption of negligence by the defendant unless the latter proves that the cause of the harm was not under their control. In other words, even when the surgery was apparently well performed, the occurrence of a complication can be a valid reason for compensating the patient if the defendant (the physician) cannot prove the actual cause was independent of their control.

In this paper, we report a case of cosmetic blepharoplasty complicated by lagophthalmos that had been wrongly related to the procedure, while it was due to missed hyperthyroidism. The study complies with the Declaration of Helsinki, and informed consent to data use was obtained.

The paper aims to discuss the importance and complexity of causal inference and informed consent in this kind of procedure.

## 2. Case Description

A 48-year-old woman underwent cosmetic blepharoplasty at a private plastic surgery clinic. On admission, she only reported autoimmune hypothyroidism (anti-Tireoglobul < 1 U/mL n.v. < 4; anti-Perossidasi 130 U/mL n.v. < 9; TSH 2.13 uU/mL n.v. 0.38–5.33; FT3 2.4 pg/mL n.v. 2.5–3.9; FT4 0.63 ng/dL n.v. 0.61–1.12), allegedly successfully treated with 100 mcg of levothyroxine + liothyronine. On clinical examination, a mild pseudogenization of the lower eyelid fat with mild blepharochalasis of the upper and lower eyelids was reported. The levator excursion was normal, and lagophthalmos, exophthalmos, and lower eyelid retraction were excluded. Informed consent, defined by the Italian Society of Plastic Surgery as involving revealing information about risks and complications, was obtained. After preoperative marking and the injection of 20 mg/mL of mepivacaine with 2% adrenaline, surgical excision of the excess skin was performed, followed by the removal of an orbicularis oculi muscle strip to expose the fat pads. The fat excision was achieved without apparent complications, and then hemostasis was carefully performed, cauterizing the cut end of the fat before releasing the hemostat. The lower eyelid blepharoplasty was approached transcutaneously; the excess skin and fat pads were carefully removed.

**Figure 1:**
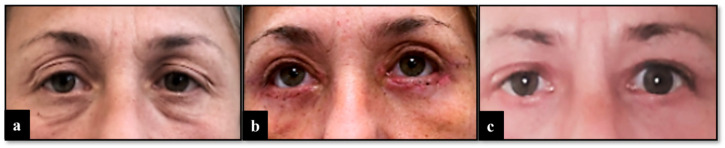
(**a**) Pre-surgery view. (**b**) View 5 days after surgery with no anomalies of interest. (**c**) View 2 months after surgery with left lagophthalmos.

**Figure 2:**
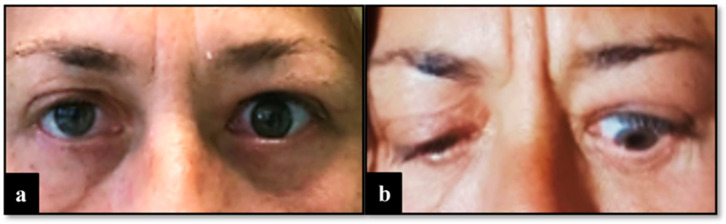
View 5 months after surgery with left lagophthalmos. (**a**) Front view with open eyes. (**b**) Front view with closed eyes.

**Figure 3:**
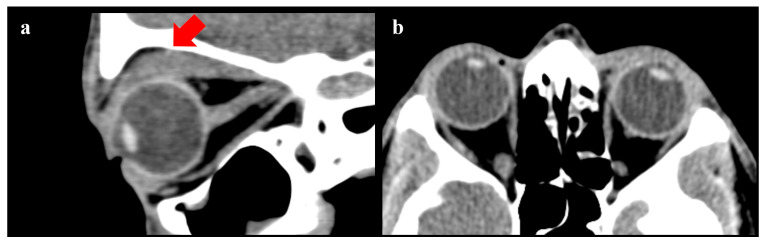
CT scan performed 5 months after surgery. (**a**) Sagittal view—the LM/SR muscle complex increase is highlighted in red. (**b**) Coronal view.

**Figure 4:**
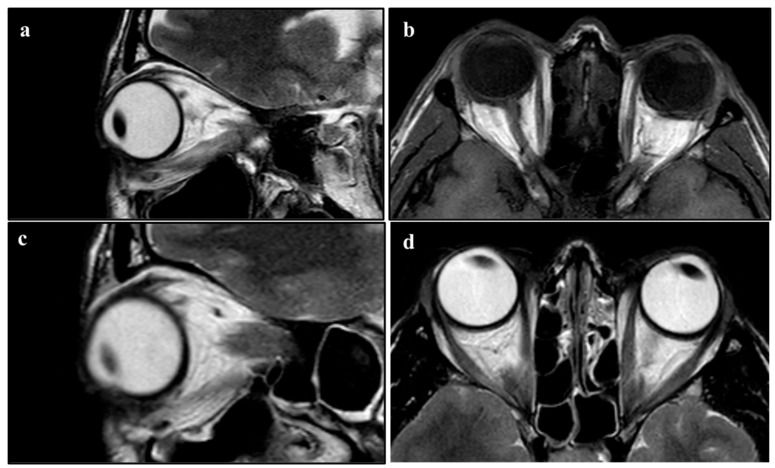
(**a**,**b**) NMR performed 10 months after surgery. (**a**) Sagittal view. (**b**) Coronal view. (**c**,**d**) NMR performed 12 months after surgery. (**c**) Sagittal view. (**d**) Coronal view.

The surgical procedure lasted 1 h, and no complications were reported. After a short period of observation, the patient was discharged. In the first two months after the surgery, no anomalies of interest occurred (Figure 1). After this period, left lagophthalmos was reported and rapidly worsened (Figure 2). Five months after the procedure, a CT scan of the head was performed to find the cause of this adverse event. It showed regular orbital fat and eye muscles and, only in the left eye, thickened eyelids (Figure 3). In the following months, MRI scans were performed, finding a bilateral increase in the thickness of the eye muscles (Figure 4).

**Figure 5:**
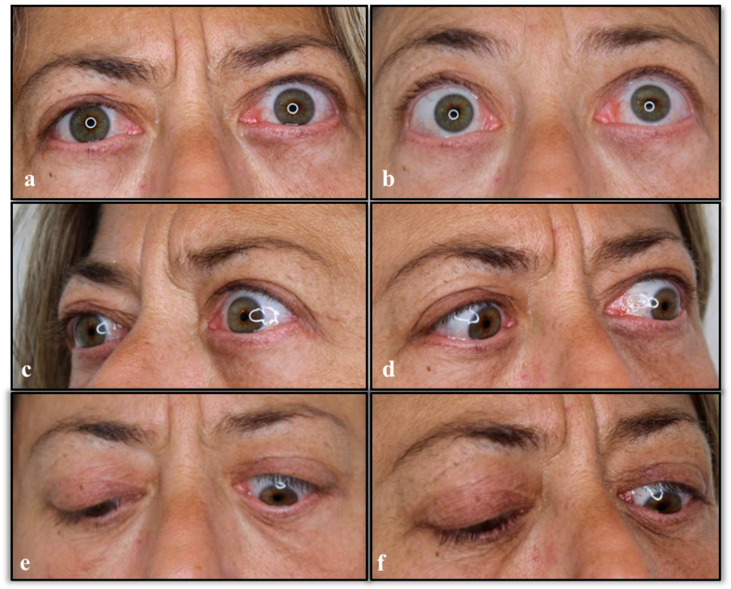
View 16 months after surgery with bilateral proptosis, asymmetrical ocular appearance with a wide lower and upper eyelid retraction in the left eye, and normal healing of blepharoplasty sutures. (**a**,**b**) Front views with open eyes. (**c**) Left side view. (**d**) Right side view. (**e**,**f**) Front views with closed eyes.

The patient claimed that the lagophthalmos was due to medical malpractice, having allegedly been caused by an over-resection of the exuberant lower eyelid tissue. Hence, 16 months after surgery, two court-appointed medical expert witnesses (a plastic surgeon and an expert in legal medicine) visited the plaintiff, finding bilateral proptosis, an asymmetrical ocular appearance with a wide lower and upper eyelid retraction in the left eye, and normal healing of the blepharoplasty sutures (Figure 5). Her ocular motility was bilaterally normal. The convergence test was negative.

Among the clinical documentation brought by the plaintiff were the results of the thyroid function tests performed for the patient over the last five years. Analyzing these documents, it was found that the reported hypothyroidism had turned into hyperthyroidism (with values of TSH < 0.01 mUI/L n.v. 0.43–3.50; FT4 2.19 ng/dL n.v. 0.70–1.48), whose diagnosis had been missed. Therefore, the lagophthalmos was assessed to have been caused by an endocrinological disorder rather than by a surgical error.

## 3. Literature Review Methodology

The review question was, “Are thyroid disfunctions usually considered contraindications to be communicated to patients who undergo blepharoplasty?”. Two investigators searched for published studies in the electronic database MEDLINE via PubMed, Embase, Scopus, Ovid, ISI Web of Science, Cochrane, and Google Scholar. Six search strings were used, combining couples of keywords using the Boolean operator AND: (1) blepharoplasty AND thyroid (51); (2) blepharoplasty and thyroid diseases (77); (3) blepharoplasty AND contraindications (21); (4) blepharoplasty AND guidelines (46), (5) blepharoplasty AND recommendations (114); (6) blepharoplasty AND consent (14). The eligibility criteria were a publication date between 1 January 1985 and 1 December 2021 and the English language. This strategy found these numbers of papers: (1) 51; (2) 77; (3) 21; (4) 46; (5) 114; (6) 14. The results were compared, and their titles and abstracts and then their full texts were examined, finding 21 eligible papers (Table 1).

## 4. Discussion

In the case we reported, post-operative lagophthalmos appeared to be due to surgical malpractice and could have led to compensation if, after a multidisciplinary and comprehensive evaluation, the cause had not been found to be an underlying (and misunderstood) thyroid disorder. Most of the claims related to surgical errors during blepharoplasty are due to complications like upper or lower eyelid malposition, lagophthalmos, corneal exposure, lacrimal system dysfunction, and dry eye syndrome [30]. However, only a small number of the claimed errors result in compensation: for instance, only 17% of cases of lagophthalmos allegedly due to errors during blepharoplasty are sentenced in favor of the plaintiff [8]. Indeed, these cases are often particularly complex from a medico-legal point of view since a negative outcome is often due to unpredictable/unavoidable factors.

In Italy, in 2017, Law 24/2017 dictated rules for assessing medical liability both in a civil and criminal context with regard to the specific clinical practice guidelines (CPGs) or recommendations enacted by scientific societies recognized by the Ministry of Health. CPGs play a dual role in medical malpractice claims and can be used in litigation by an accused physician as a defense (exculpatory evidence) and by patients alleging a breach of the standard of care (inculpatory evidence).

Establishing a breach in the standard of care is key to medical malpractice claims under the negligence standard, in which a defendant physician attempts to assert that he or she has complied with the standard of care, and a plaintiff conversely contends the acceptable standard was not met [31,32].

The duty of care entails both a correct procedure from a technical point of view and correct information concerning the post-operative risks. Therefore, a surgeon could be found liable for having failed to comply with one of or both these aspects of their duty [33].

In the case we reported, the lagophthalmos was not due to a technical error but to the worsening of hyperthyroidism, whose diagnosis was missed. This occurrence is of significant medico-legal interest since, in civil law, the standard of proof adopts the principle of the so-called “preponderance of the evidence”, and thus post-operative lagophthalmos—which, as previously stated, is one of the commonest complications of the procedure—is usually interpreted as an indicator of a surgical error. An important criterion to determine whether there is liability is to compare the conduct of the defendant with the standard of care established by the guidelines and the evidence-based best practices [31,32,33,34].

According to the reviewed literature, thyroid disorders do not contraindicate (cosmetic) blepharoplasty [20,24]. However, the current evidence indicates that thyroid function should always be screened before blepharoplasty and, in the case of disorders, a stabilization of function for at least 6–12 months before surgery and an eye examination with the evaluation of the periocular muscles are highly recommended [25,35,36].

Generally speaking, no guidelines or scientific literature contraindicate blepharoplasty for patients affected by thyroid diseases, even if cosmetic blepharoplasty might be deemed inappropriate for patients affected by both hypothyroidism and hyperthyroidism. The correct balance of thyroid function before surgery does not represent an absolute index of stability of thyroid function. Especially in cases of autoimmune thyroiditis, as in the case of this patient, in a low percentage of cases, there can be a rapid change from hypothyroidism and euthyroidism to hyperthyroidism.

Uneven guidelines on blepharoplasty for functional or cosmetic reasons and major evidence on patients affected by thyroid dysfunction jeopardize clinical procedures and increase the risk of litigation.

Therefore, from a medico-legal point of view, there are two arguments to be enforced to avoid undeserved compensations: i) informed consent should always clearly represent the risk that, in the case of thyroid disfunctions, after the surgery, complications not due to malpractice could occur; ii) the guidelines for cosmetic and functional blepharoplasty should strongly recommend endocrinological consultation in patients at risk (e.g., female patients with a known history of thyroid disease).

## Figures and Tables

**Table 1:** Results of Literature Review.

Author	Article Type	Indications/Contraindications to Blepharoplasty in Patients with Thyroid Conditions *
Holt J.E. et al. (1985) [9]	Classical article	*“…When performing surgery in and around the eyelids, a complete ophthalmologic examination should be performed on every patient. This includes documentation of visual acuity, pupillary function, intraocular pressure, extraocular muscle balance, and ophthalmoscopic examination…”*
Klatsky S.A. et al. (1992) [10]	Classical article	*“…It is important when evaluating patients requesting cosmetic surgery that medical conditions simulating aging changes be considered…”*
Fulton J.E. (1999) [11]	Classical article	*“... After reviewing the history of the orbital area, the general health should be examined. Are there any systemic illnesses such as multiple sclerosis, **thyroid imbalance**, hypertension or diabetes?...”*
Rosenthal E.L. et al. (1999) [12]	Case report	*“… Given the development of symptoms in the immediate postoperative period, it is likely that blepharoplasty activated a previously subclinical GO* [ed. GO: Graves Orbitopathy] *…This is the first report of a patient with subclinical Graves disease developing GO after blepharoplasty. Although extremely rare, this case underscores the importance of preoperative screening for a previous history of thyroid or orbital diseases…”*
Rohrich R.J. et al. (2004) [13]	Classical article	*“...Medical and ophthalmologic histories must be obtained from the patient, including any history of chronic illness, hypertension, diabetes, cardiac disease, bleeding disorders, **thyroid disturbances**, or surgery...”*
American Society of Plastic Surgeons (ASPS) (2007) [14]	Practice guidelines	*“...CONSULTATION: Preoperative consultation should evaluate the patient’s reasons for seeking surgery. Patients present with a variety of symptoms or combination of symptoms including edema, … dry eye, medications, allergies, history of eyelid swelling, **thyroid disease**, heart failure, and bleeding tendencies…”*
Trussler A.P. et al. (2008) [15]	Review	*“...Preoperative patient evaluation for blepharoplasty should document medical and ophthalmologic histories.^1^ This should include lifestyle history (smoking, exercise tolerance, and alcohol use), history of chronic illnesses, hypertension, diabetes, cardiac disease, bleeding and/or clotting disorders, **thyroid disturbances**, or previous operations. Medications, including aspirin and other anticoagulants, should be listed and withheld for at least 2 weeks preoperative...”*
Naik M.N. et al. (2009) [16]	Review	*“...Preoperative patient evaluation for blepharoplasty should document medical and ophthalmologic history such as chronic systemic diseases and medications. Ophthalmologic history should be obtained, including vision, corrective lenses, trauma, glaucoma, allergic reactions, excess tearing, and dry eyes…”*
Friedland J.A. et al. (2010) [17]	Review	*“...Preoperative patient evaluation for blepharoplasty should include general medical and periorbital histories…bleeding and/or clotting disorders, **thyroid problems**, and chronic illnesses, such as diabetes and hypertension, should be documented…”*
Drolet B.C. et al. (2014) [18]	Review	*“...All patients require a thorough medical history. Comorbidities should be noted, including hypertension, diabetes mellitus, bleeding disorders, and **thyroid disease**, along with significant chronic illnesses that would preclude an aesthetic operation…A detailed ophthalmologic history, including prior trauma or surgery, visual disturbances, and dry eyes, should be obtained…”*
Custer P.L. (2014) [19]	Book chapter	*“...Patients with the following conditions may be **at higher risk for complications** following blepharoplasty or require specialized surgical techniques: unrealistic expectations, prior eyelid/facial surgery, dry eye symptoms, **thyroid disease**, prominent eyes, marked orbital asymmetry, significant coexisting medical problems...”*
Italian Association of Aesthetics and Plastic Surgery (AICPE) (2015) [20]	Guidelines	*“…it is preferable to avoid surgery in case of: alterations in coagulation, hypertension or precarious general conditions... if there is any doubt about ocular pathology, it is advisable to have an ophthalmological examination. Blood tests are recommended: blood count, PT, PTT and glycaemia…”*
Italian Society of Reconstructive and Aesthetic Plastic Surgery (SICPRE) (2015) [21]	Information from guidelines	*“…Before proceeding with corrective blepharoplasty, the patient may be advised to undergo an eye examination which must include the determination of the visual field and the measurement of the ocular tone. **Thyroid function screening tests may be required in special cases and especially for women**…“*
Scawn R. et al. (2016) [22]	Classical article	*“…Before considering blepharoplasty, potential contraindications to surgery should be elucidated; these include patients with psychological issues, dry eyes, active inflammatory cicatrising skin conditions such as eczema and psoriasis and multi-revision surgeries…”*
Bhattacharjee K. et al. (2017) [23]	Review	*“...complete medical and ocular history should be obtained, along with a thorough ophthalmologic examination. A proper history of any trauma or previous surgery should be recorded. **Patients should be evaluated for thyroid disease** and dry eye disease. Seventh nerve function should also be evaluated...”.*
Joseph A. et al. (2018) [24]	Book chapter	*“…**Eyelid retraction, lid lag on downgaze and lagophthalmos, and decreased convergence may be found in patients with thyroid-related orbitopathy. Blepharoplasty should be reserved for patients with quiescent disease and undertaken after any proptosis, motility dysfunction, and eyelid retraction have stabilized for 6 months to 1 year or have been definitively treated. In patients with suspected thyroid eye disease, referral to an endocrinologist or internist may be necessary for appropriate systemic workup, including serum thyroid hormone levels (triiodothyronine [T3], levorotatory thyroxine [T4], thyroid stimulating hormone [TSH]). Orbital computed tomography may demonstrate enlargement of the extraocular muscles and increased orbital fat when systemic signs are completely lacking early in the course of the disease**…”*
MassHealth (2019) [25]	Guidelines	*“...Any related disease process, such as myasthenia gravis or **thyroid condition** or oculomotor nerve palsy, **is documented as stable and with optimal medical management prior to the consideration of surgery**;...”*
Kim K. et al. (2019) [26]	Guideline draft	*“...The initial patient evaluation should include general medical and periorbital history. A detailed medical and focused history should document elements of previous eye and eyelid surgery, cardiac and chronic illness, bleeding disorders, medications and smoking…A physical examination should be performed. The eye examination should consist of basic visual acuity, extraocular muscle and pupil evaluation, and Bell’s phenomenon for corneal protection…”*
Caughlin B.P. (2020) [27]	Book chapter	*“…Relative contraindications to blepharoplasty: Actinic changes, acne rosacea, keloids, herpes zoster, **thyroid abnormalities**, autoimmune diseases, smoking history, extensive history of dry eye syndrome, and acute angle glaucoma history. Generally, should wait approximately 6 months after LASIK or PRK (exact timing varies depending on the source)…”*
Kwitko G.M. et al. (2021) [28]	Review	*“...Most contraindications for ptosis surgery revolve around the exposure of the cornea. Conditions like **thyroid orbitopathy**, progressive external ophthalmoplegia, or loss of Bell’s phenomenon can make patients more prone to exposure keratopathy after ptosis surgery: **a more conservative approach is needed in these patients**…**CT scan of the orbits should be obtained if an orbital process such as thyroid orbitopathy** or an orbital tumor is suspected. Slit lamp evaluation is essential to detect corneal erosions or dry eye…”*
Rebowe R.E. et al. (2021) [29]	Review	*“…All patients undergoing eyelid procedures should be questioned regarding ophthalmologic pathology and should receive a full eye exam, complete with the retinal examination. Specifically, patients should be questioned regarding preoperative visual acuity, symptoms of dry eyes, and visual obstruction. Furthermore, the full medical history should include pathology related to systemic disease with ophthalmologic manifestations, including **thyroid disease**, diabetes, hypertension, or inflammatory diseases treated with steroids. A history of bleeding or clotting disorders should also be elicited…)*

* Specific mentions of thyroid disturbances are highlighted in bold.

## Data Availability

All relevant data are provided in the paper.
